# Feasibility and comparison of 3D modified rosette ultra-short echo time (PETALUTE) with conventional weighted acquisition in ^31^P-MRSI

**DOI:** 10.1038/s41598-025-90630-y

**Published:** 2025-02-22

**Authors:** Brian Bozymski, Xin Shen, Ali Özen, Mark Chiew, M. Albert Thomas, William T. Clarke, Stephen Sawiak, Ulrike Dydak, Uzay Emir

**Affiliations:** 1 School of Health Sciences, Purdue University, West Lafayette, IN USA; 2 Radiology and Biomedical Imaging, University of California San Francisco, San Francisco, CA USA; 3 Weldon School of Biomedical Engineering, Purdue University, West Lafayette, IN USA; 4 Division of Medical Physics, Department of Radiology, University Medical Center Freiburg, University of Freiburg, Freiburg, Germany; 5 Nuffield Department of Clinical Neurosciences, Wellcome Centre for Integrative Neuroimaging, FMRIB, University of Oxford, Oxford, UK; 6 Department of Medical Biophysics, University of Toronto, Toronto, Canada; 7 Department of Radiology, University of California, Los Angeles, CA USA; 8 Department of Clinical Neuroscience, University of Cambridge, Cambridge, UK; 9 Department of Physiology, Development and Neuroscience, University of Cambridge, Cambridge, UK; 10 Department of Radiology and Imaging Sciences, Indiana University School of Medicine, Indianapolis, IN USA; 11 Department of Radiology, University of North Carolina, Chapel Hill, NC USA; 12 the Lampe Joint Department of Biomedical Engineering , University of North Carolina, NC Chapel Hill, USA

**Keywords:** Magnetic resonance imaging, Translational research

## Abstract

Phosphorus-31 magnetic resonance spectroscopic imaging (^31^P-MRSI) provides valuable non-invasivein vivoinformation on tissue metabolism but is burdened by poor sensitivity and prolonged scan duration. Ultra-short echo time (UTE) acquisitions minimize signal loss when probing signals with relatively short spin-spin relaxation time (T_2_), while also preventing first-order dephasing. Here, a three-dimensional (3D) UTE sequence with a rosette k-space trajectory (PETALUTE) is applied to ^31^P-MRSI at 3T. Conventional weighted MRSI employs highly regular Cartesian k-space sampling, susceptible to substantial artifacts when accelerated via undersampling. In contrast, this novel sequence’s “petal-like” pattern offers incoherent sampling more suitable for compressed sensing (CS). These results showcase the competitive performance of PETALUTE against conventional weighted ^31^P-MRSI with simulation, phantom, and in vivo leg muscle comparisons.

**Supplementary Information:**

The online version contains supplementary material available at 10.1038/s41598-025-90630-y.

## Introduction

Phosphorous-31 magnetic resonance spectroscopy ^31^P-MRS), the longest-standing in vivo MRS modality, can be an invaluable tool for probing in vivo metabolites such as phosphocreatine (PCr), inorganic phosphate (Pi), phosphomonoesters (PMEs), phosphodiesters (PDEs), and adenosine triphosphate (ATP)^1,2^. As fundamental phospholipids and constituents of the high-energy phosphate pathway, these ^31^P metabolites provide noninvasive measures of tissue pH, lipid metabolism, and oxidative bioenergetics^3,4^. Thus ^31^, P-MRS possesses versatile diagnostic and prognostic potential. For instance, elevated PME/PDE ratios and reduced ATP levels have been reported in diseased and cancerous liver tissue, often correlated with classical plasma markers and Child-Pugh scores^5–9^. Furthermore ^31^, P-MRS has been used to assess whole-liver treatment efficacy, monitoring metabolite changes in malignant tissues following therapy^10^. Likewise, diminished PCr/ATP ratios and post-exercise PCr recovery rates have been measured in cardiac and skeletal muscles of patients with type 2 diabetes^11^. Numerous endeavors have employed ^31^P-MRS in the brain, heart, and muscle, seeking out alterations in neurodegenerative, cardiovascular, metabolic, and oncological diseases^12–20^.

While relevant^1^H-MRS metabolites can be obscured by contaminating fat, water, and macromolecular background signals, widely spaced ^31^P spectral peaks are more easily elucidated due to the absence of these nuisance signals. However, in contrast to^1^H-MRS ^31^, P-MRS is burdened by a lower gyromagnetic ratio and relatively short spin-spin metabolite relaxation times (T_2_);^21,22^ these factors engender extremely poor in vivo relative sensitivity and force a delicate balance between SNR, resolution, and scan duration. Low ^31^P-MRS tissue concentrations (approximately 2 mM γ-ATP in liver^23^) further exacerbate SNR challenges, so that commonly used acquisition delays (T_E_ > 300 µs) with conventional methods result in prolonged acquisition, phase distortions, baseline roll, and subsequent operator errors during metabolite quantification. Such complications have been severely limiting factors in the clinical feasibility of ^31^P-MRS. Recent advances in coil engineering and the introduction of ultra-high field (UHF, B_0_ > 3T) scanners have assisted in mitigating these limiting factors; one experiment demonstrated a 2.8-factor increase in PCr SNR at 7T relative to 3T^24^. Conversely, UHF acquisitions also necessitate larger spectral bandwidth (SBW), with a 40-ppm range requiring approximately 2.0 kHz at 3T but 4.8 kHz at 7T. Still, excessive acquisition durations remain the clear barrier to clinical translation without innovative acceleration.

**Fig. 1:**
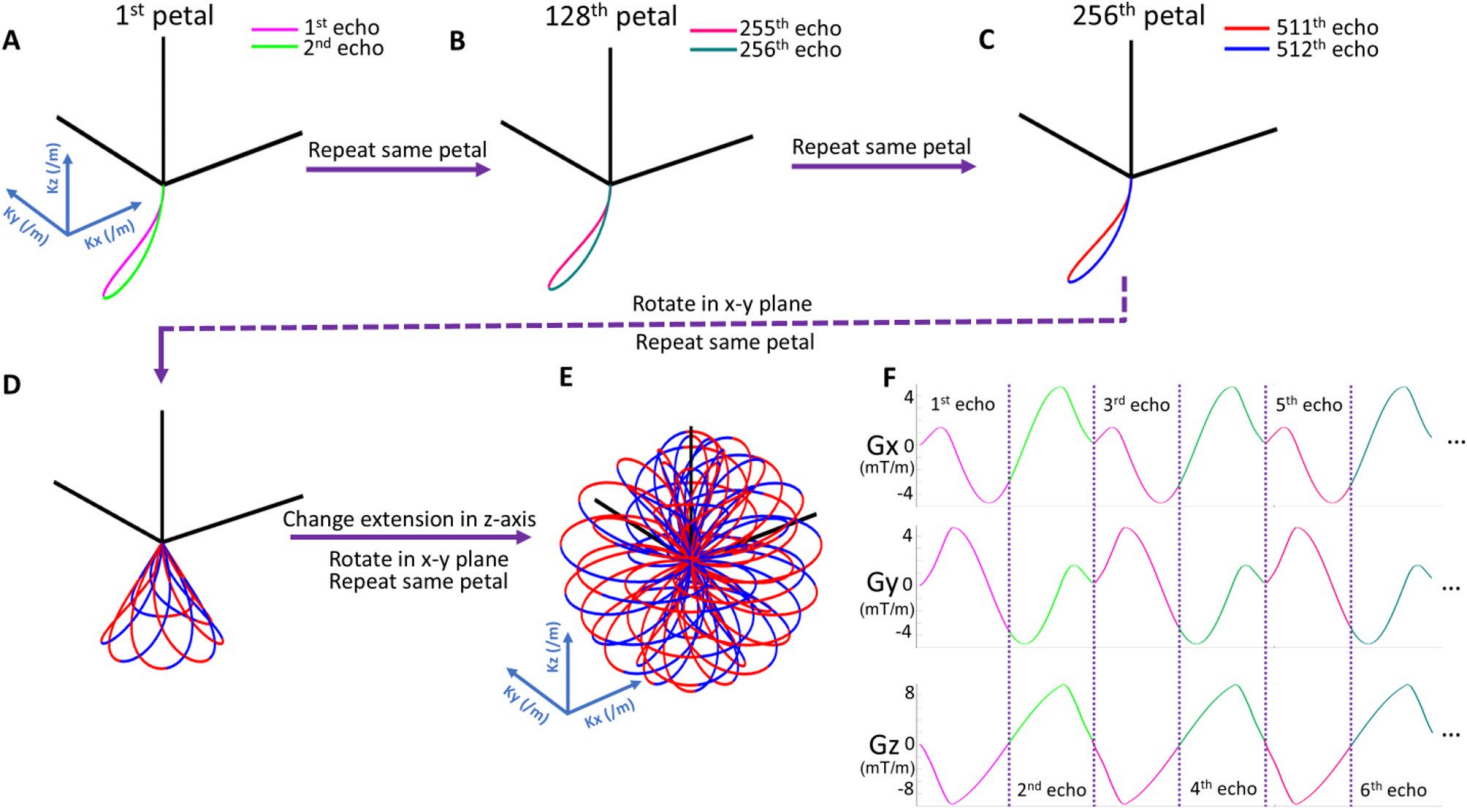
Illustration of 3D rosette k-space trajectory and gradients. (**A**)–(**C**) Acquisition begins at k-space center for every petal, crossing k-space origin twice at each petal’s beginning and end. Petals can be manually separated into two halves, similar to odd and even echoes in EPSI MRSI. (**D**,**E**) Varied petal rotations form the rosette pattern, providing sufficient k-space coverage. (**F**) With the closed-loop trajectory, acquisition delay is further minimized by enabling the analog to digital converter (ADC) for sampling during gradient ramp-up.

To address these points, we propose a novel three-dimensional (3D), ultra-short echo time (UTE) sequence with a rosette^25^ k-space trajectory (PETALUTE, previously validated in ultra-short-T_2_ imaging^26,27^, brain iron mapping^28,29^, and sodium quantification^30^) for ^31^P magnetic resonance spectroscopic imaging (MRSI)^31^. Compared to conventional Cartesian MRSI k-space trajectories, rosette’s “petal-like” pattern (Fig. 1) maps 3D k-space far more efficiently. Additionally, rosette’s relatively incoherent data sampling allows the possibility of significant acceleration through higher undersampling factors and compressed sensing (CS) reconstruction; offering better k-space coverage when compared to radial and spiral trajectories, generalized rosette’s curvature affords superior SNR performance under aggressive acceleration^32^. Furthermore, the rosette’s center-out k-space design enables UTE acquisition, thereby permitting capture of short-T_2_ signals before significant transverse signal decay and first-order dephasing occur; this enhances SNR, simplifies spectral pre-processing, and minimizes operator-dependent quantification errors.

Substantial efforts have been invested towards clinically feasible ^31^P-MRSI, experimenting with short repetition times (T_R_), measuring multiple k-space points per T_R_, k-space undersampling, enhancing reconstruction via prior knowledge, and their conceivable combinations^33^. 3D extensions of ISIS have shown promise in UHF preclinical and 3T cardiac studies but remain limited by time resolution and motion artifact sensitivity^34,35^. Non-localized or FID acquisitions are often preferred to minimize rapid ^31^P metabolite T_2_-decay, but also to overcome specific absorption rate (SAR) limitations at UHFs. Thus, variations of spatial-spectral encoding (SSE) schemes and their synergies with k-space undersampling appear to be the more promising avenue forward; several Cartesian and non-Cartesian acquisition designs offer varying degrees of SNR efficiency, k-space weighting, gradient system demands, and undersampling acceleration potential.

For example, flyback and multishot EPSI have been tested in brain^36,37^ and skeletal calf muscle^38^, offering considerable time savings over conventional phase encoding when combined with CS acceleration;^39^ despite its acceleration potential, EPSI^36,40–42^ offers lower SNR efficiency and SBW at fine resolutions than other SSE options. Density-weighted concentric ring^43,44^ trajectories (CRTs) grant increased SNR efficiency and SBW limits, enabling faster MRSI even with UHF systems. CRTs boast flexibility in weighting and temporal interleaves, allowing tailoring according to acceleration needs and gradient slew rates. Similarly, spiral-encoded^45^^31^P-MRSI has exhibited faster dynamic calf muscle mapping than conventional elliptical phase-encoded acquisition^46^. Though spirals offer high acceleration, SNR efficiency, and customizable weighting, they are also limited by SBW and gradient system hardware.

Conventional MRSI FIDs commonly possess T_E_ on the order of 1–2 ms, restricted by the duration of constant excitation pulses and phase encoding gradients to reach the outermost k-space points. This acquisition delay can be decreased using variable pulse widths and amplitudes. Sampling throughout gradient transition periods, or ramp sampling, remains an additional option for minimizing T_E_. Studies have demonstrated T_E_ as low as 480 µs and 520 µs for EPSI and radial EPSI, respectively^39,47^. Acquisitions using such UTE-MRSI techniques^48^ have achieved T_E_ = 300 µs at 3T and T_E_ = 500 µs at 7T^49,50^. In non-Cartesian center-out k-space trajectories employing ramp sampling, T_E_ is primarily limited by the dead time between coil transmit-receive switching, permitting the shortest possible T_E_. Despite this possibility, SBW constraints and conventional sequence parameter (T_A_/FOV) matching have prevented extensive investigation of non-Cartesian UTE ^31^P-MRSI.

In this study, we evaluate the PETALUTE ^31^P-MRSI with a novel rosette k-space trajectory by comparing its performance to conventional 3D weighted Cartesian ^31^P-MRSI in the quadriceps muscle at 3T. We ultimately aim to demonstrate its potential value in clinical spectroscopic acquisitions.

## Methods

### k-space trajectory designs for MRSI

#### PETALUTE

General sequence parameters were as follows: T_A_ = 36:00, T_R_ = 350 ms, T_E_ = 65 µs, matrix size ($\:{N}_{x}{\times\:N}_{y}\times\:{N}_{z}$) = 24$\:\times\:$24$\:\times\:$24, nominal voxel size = 8 mL, FOV = 480$\:\times\:$480$\:\times\:$480 mm^3^, SBW = 2083 Hz, time samples = 512. Parameters are summarized in Table 1.

**Table 1:** Table of protocol parameters for conventional 3D weighted MRSI and novel PETALUTE MRSI acquisitions. Nominal voxel size was matched between methods, with total acquisition time approximately equal between the two sequences. SBWs were matched via interpolation during post-processing.

	PETALUTE	Weighted MRSI
T_A_ (mm: ss)	36:00	36:56
T_R_	350 ms	1000 ms
T_E_	70 µs	2.3 ms
α	20°	90°
Number of averages	4	4
Reconstruction matrix	24	16
Nominal voxel (mL)	8	8
FOV (mm^3^)	480	320
Bandwidth (Hz)	2083	2200
Time samples	512	512
T_A_ relative to MRSI	0.97	1.00

As in prior work^26^, 3D PETALUTE k-space trajectory (Fig. 1) for ^31^P-MRSI was generated with Eqs. (1) and (2):

1 $$\begin{aligned} K_{{xy}} & = Kx\left( t \right) + iKy\left( t \right) \\ & = \left( {K~{\text{max}}*\cos \left( \varphi \right)} \right)\sin \left( {\omega _{1} t} \right)e^{{i\omega _{2} t + \beta }} ~ \\ \end{aligned}$$

2 $$K_{z} \left( t \right) = (K~{\text{max}}*\sin \left( \varphi \right))\sin \left( {\omega _{1} t} \right)~$$

Where *K* max is the furthest distance in k-space (= 25 cycle/m in this study), the radial direction oscillation frequency ($\:{\omega\:}_{1}$) and the angular direction oscillation frequency ($\:{\omega\:}_{2}$) were set equally ($\:{{\omega\:}_{1}=\omega\:}_{2}$), $\:\phi\:$ determines the z-axis location, which was sampled uniformly in the range of [-$\:\pi\:$/2, $\:\pi\:$/2], and $\:\beta\:$ determines the initial angular direction phase, which was sampled uniformly in the range of [0, 2$\:\pi\:$].

$$\:K\text{max}=\frac{{N}_{x}}{2*\text{F}\text{O}\text{V}}=25\:\text{c}\text{y}\text{c}\text{l}\text{e}/\text{m}\:$$

Other imaging parameters included: hard rectangular RF pulse duration = 50 µs, flip angle α = 20°, dwell time Δt = 5 µs in the readout, and each rosette petal designed with *N*_*pp*_ = 96 points. This leads to

$$\:\text{S}\text{B}\text{W}=\frac{1}{{N}_{pp}\text{*}{\Delta\:}\text{t}}={\left(480\:{\upmu\:}\text{s}\right)}^{-1}=2083\:\text{H}\text{z}$$

as well as

$$\:{\omega\:}_{1}={\omega\:}_{2}=\pi\:*\text{S}\text{B}\text{W}=6545\:\text{r}\text{a}\text{d}/\text{s}$$

and the resulting − 20 to + 20 ppm in the 3T spectral range is more than sufficient for ^31^P-MRS. In reconstruction, each petal was downsampled to *N*_*pp*_ = 48 by averaging the oversampled points. With a matrix size of 24$\:\times\:$24$\:\times\:$24 for the reconstruction, the required number of petals (*N*_*p*_) to satisfy Nyquist criterion was calculated as

$$\:{N}_{p}=4\pi\:*{\left(\frac{{N}_{x}}{2}\right)}^{2}\approx\:1810$$

However, due to the rosette’s efficient sampling scheme, only 79.8% coverage (i.e., *N*_*p*_ = 1444) was defined as full k-space acquisition. Thus, the acquisition time per average was calculated as

$$\:{\text{T}}_{\text{A}}={N}_{p}*{\text{T}}_{\text{R}}=505\:\text{s}$$

or roughly 9 min. A complete description including the influence of trajectory parameters, Nyquist criterion, and the specific gradient ramp-up of this 3D rosette k-space pattern is provided in earlier work (Shen et al.)^26^.

#### Conventional weighted MRSI

Conventional Cartesian 3D acquisitions used the vendor-provided ^31^P-MRSI FID with k-space weighting and Hanning filter. Sequence parameters were as follows: T_A_ = 36:56, T_R_ = 1000 ms, T_E_ = 2.3 ms, matrix size ($\:{N}_{x}\times\:{N}_{y}\times\:{N}_{z}$) = 16$\:\times\:$16$\:\times\:$16, nominal voxel size = 8 mL, FOV = 320$\:\times\:$320$\:\times\:$320 mm^3^, SBW = 2200 Hz, time samples = 512. The acquisition used the vendor H-Sinc excitation pulse with 1.28 ms duration and α = 90°.

Parameters are summarized in Table 1.

### Simulations

To assess the theoretical performance of the PETALUTE relative to conventional weighted MRSI, MATLAB (MathWorks, Natick, USA) simulations were run examining side lobes and SNR relative to the spatial response function (SRF). A simple, constant 3D object was placed at the origin of a 48$\:\times\:$48$\:\times\:$48 grid (FOV = 480 mm isotropic producing a nominal voxel size = 1 mL) and reconstructed using the non-uniform FFT (NUFFT) method^51^ and k-space information for each in vivo acquisition. We discuss both SRF and point spread function (PSF) since the former specifically estimates side lobes and signal bleed between adjacent voxels, while the latter measures contribution from a single object point to the entire population of voxels.

### Experimental comparison

All data acquisition occurred on a 3T MRI system (Prisma, Siemens, Erlangen, Germany) with G_max_ = 80 mT/m and slew rate = 200 mT/m/ms isotropic. This study conforms to the Declaration of Helsinki, with all protocols approved and conducted in accordance with the Institutional Review Boards of Purdue University. Written informed consent was obtained from all human subjects.

The rosette and conventional acquisitions were tested with a uniform 2-liter bottle phantom (0.17 mg/mL phosphoric acid) using a dual-tuned ^1^H/^31^P Tx/Rx flexible 11-cm surface coil (RAPID Biomedical). For in vivo comparison, five healthy volunteers (BMI = 26 ± 2 kg/m^2^; age = 29 ± 5 years; 2 f / 3 m) received leg scans with an 8-channel, dual-tuned ^1^H/^31^P Tx/Rx phased array coil^52^ (Stark Contrast, Erlangen, Germany). Quadriceps was chosen for its superior PCr SNR and absence of respiratory motion during prolonged scanning. Subjects were positioned feet-first and supine, with the upper quadriceps tightly surrounded by the coil plates. Following localizer imaging, the adjustment volume was manually positioned (spanning both legs), and linewidth was minimized using a 3D GRE field map and interactive Siemens shimming. Each subject was scanned first with the conventional weighted Cartesian acquisition followed uninterrupted by the PETALUTE ^31^P-MRSI.

### Post-processing and reconstruction

Raw data files were exported for reconstruction and pre-processing in MATLAB. Gridding and FFT were completed using adjoint, type 2 (forward) NUFFT regridding^53^ and density compensation according to the Pipe method^54^. Data were Hanning filtered and, when necessary, coil-combined using whitened singular value decomposition (wSVD) with the noise covariance matrix (Σ) approximated from spectral regions without signal^55^. Spectra from the PETALUTE were zero-order phased by maximizing the integral of the largest peak (PCr, 0 ppm). Spectra from the conventional weighted MRSI were both zero-order phased and first-order phased to correct a 2.3-ms delay.

### SNR and quantification

Spectra were fitted within the Oxford Spectroscopy Analysis (OXSA) toolbox^56^ using AMARES methods. Metabolite peak SNRs were calculated from real-valued spectra according to Eq. (3), with noise variance calculated from a residual region lacking metabolite signals. As an additional signal quantification metric, “raw SNR” (Eq. (4)) was estimated by dividing the highest absolute-value peak point by the noise variance in an off-spectrum region; this method carries the advantage of consistently assessing signal strength regardless of any interfering spectral phase.

3 $${\text{SNR}}_{{{\text{OXSA}}}} = \frac{{{\text{Peak~Signal~Fit~}}\left( {{\text{Real}}} \right)}}{{{\text{RMS}}_{{{\text{residual~noise}}}} }}$$

4 $${\text{SNR}}_{{{\text{raw}}}} = \frac{{{\text{Maximum~Absolute~Peak~Amplitude}}}}{{{\text{RMS}}_{{{\text{off}} - {\text{spectrum~noise}}}} }}$$

**Fig. 2:**
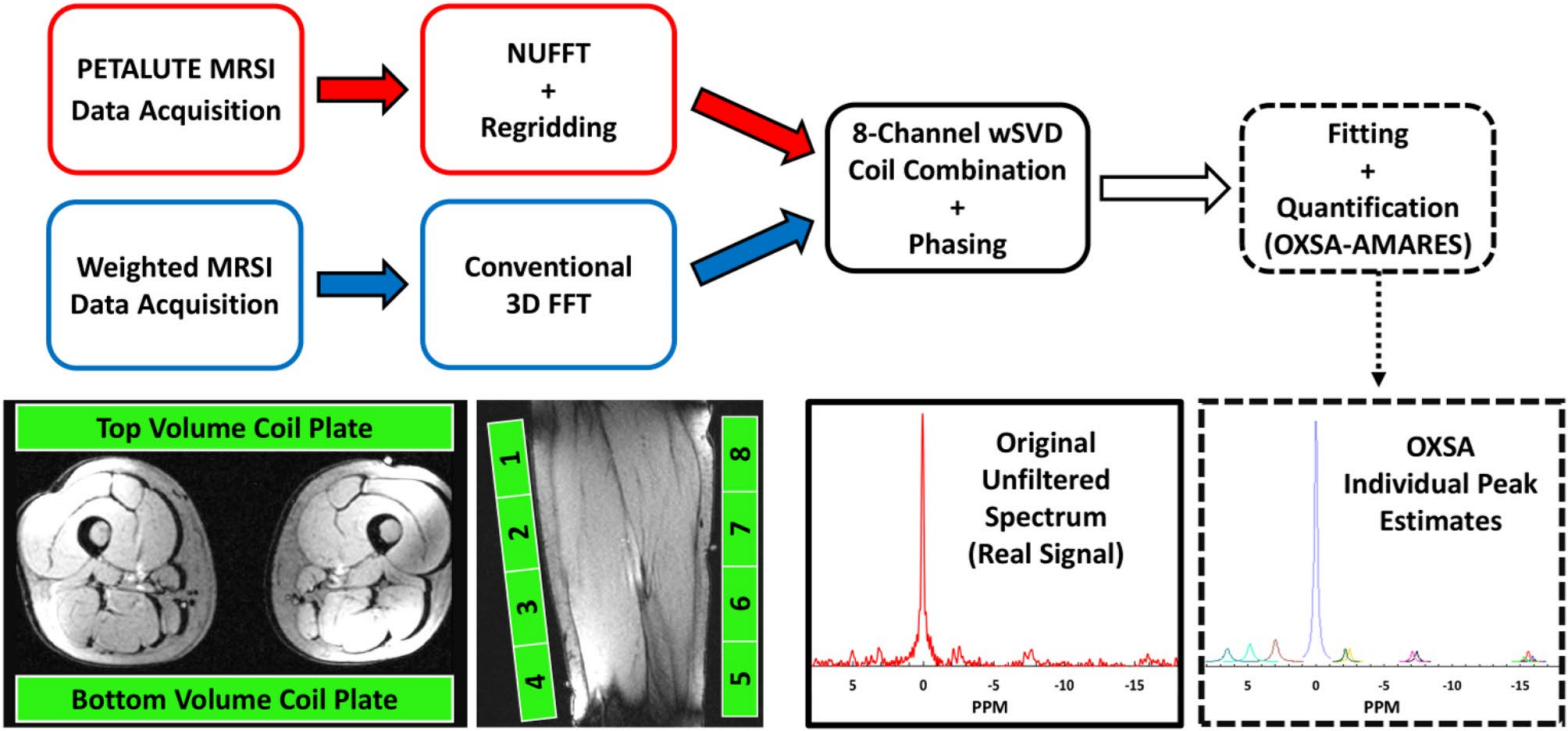
Workflow of data acquisition, reconstruction, processing, and analysis. Subjects were positioned feet-first supine with both quadriceps positioned between the 30-cm phased array coil plates. Raw data were exported, appropriately reconstructed, coil-combined, and phased prior to fitting and quantification.

**Fig. 3:**
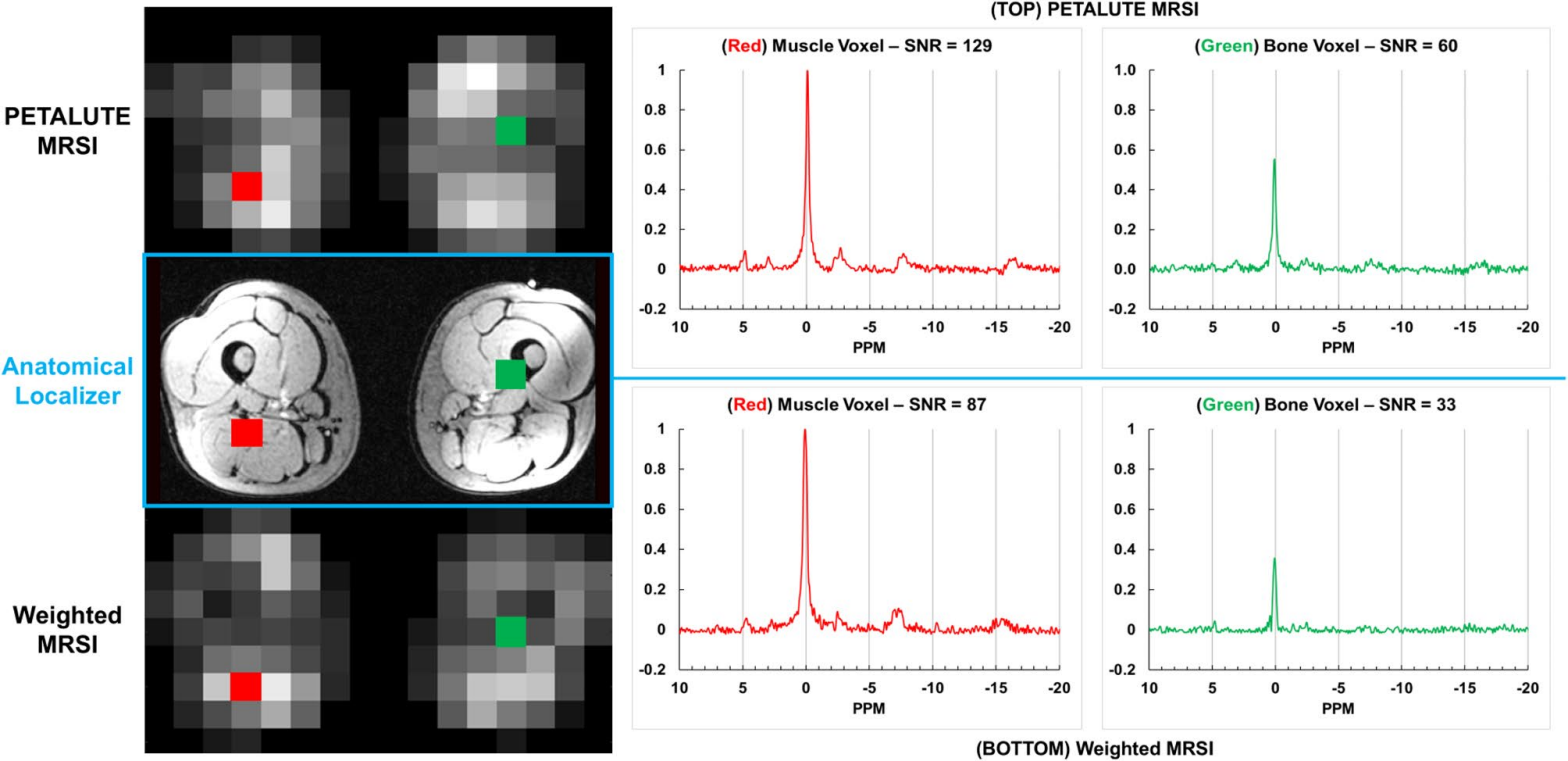
(**A**) Signal intensity and selected central axial slices for Subject 3’s PETALUTE acquisition. (**B**) Visualization of “raw SNR” calculation on ^31^P-MRS muscle spectrum in one voxel. (**C**) Results for quantifiable (SNR > 3) voxels within selection.

Figures 2 and 3 summarize data acquisition, reconstruction, processing, and analysis workflow.

### Quantitative analysis

The performance of PETALUTE and weighted MRSI in phantom solution and quadriceps muscle were assessed using Pi and PCr metabolite signals, respectively. Quantification considered the central, highest signal axial slices within each subject, attempting to quantify every voxel. Only voxels with SNR > 3 and OXSA-AMARES Cramér-Rao lower bound (CRLB) goodness of fit smaller than 20% for PCr peak were included in the final analysis. The statistical significance of SNR results was assessed using right-tailed Welch’s t-tests.

## Results

### Spatial response function simulation comparison of PETALUTE with weighted MRSI

**Fig. 4:**
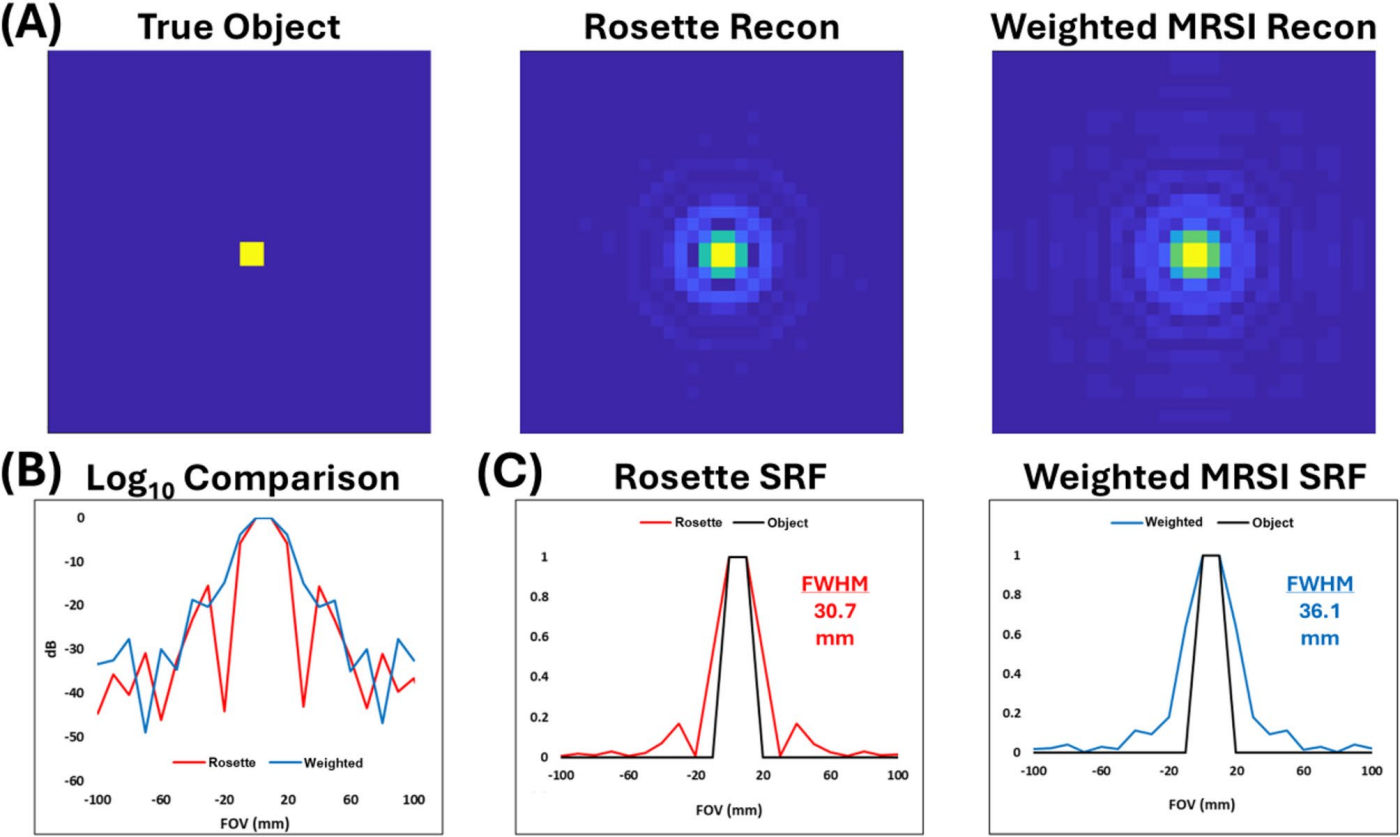
Results of spatial response function (SRF) simulation for novel 3D PETALUTE and conventional 3D weighted MRSI sequences. (**A**) 2D (xy-plane) SRFs for simulated object and each k-space trajectory at center of the FOV. (**B**) 1D (x-axis) log_10_ decibel comparison between k-space trajectories at center of the FOV. (**C**) 1D (x-axis) comparisons between each normalized reconstruction and the true simulated object at center of the FOV.

The impact of varying k-space sampling trajectories on image quality can be evaluated via SRF simulations as shown in Fig. 4. FWHMs along the x-axis at the center of the FOV were comparable between rosette (30.7 mm) and weighted MRSI (36.1 mm). Both acquisition schemes exhibit noticeable sidelobe noise, albeit with slightly reduced side lobes in the rosette trajectory close to the object origin.

### Phantom comparison of PETALUTE with weighted MRSI

**Fig. 5:**
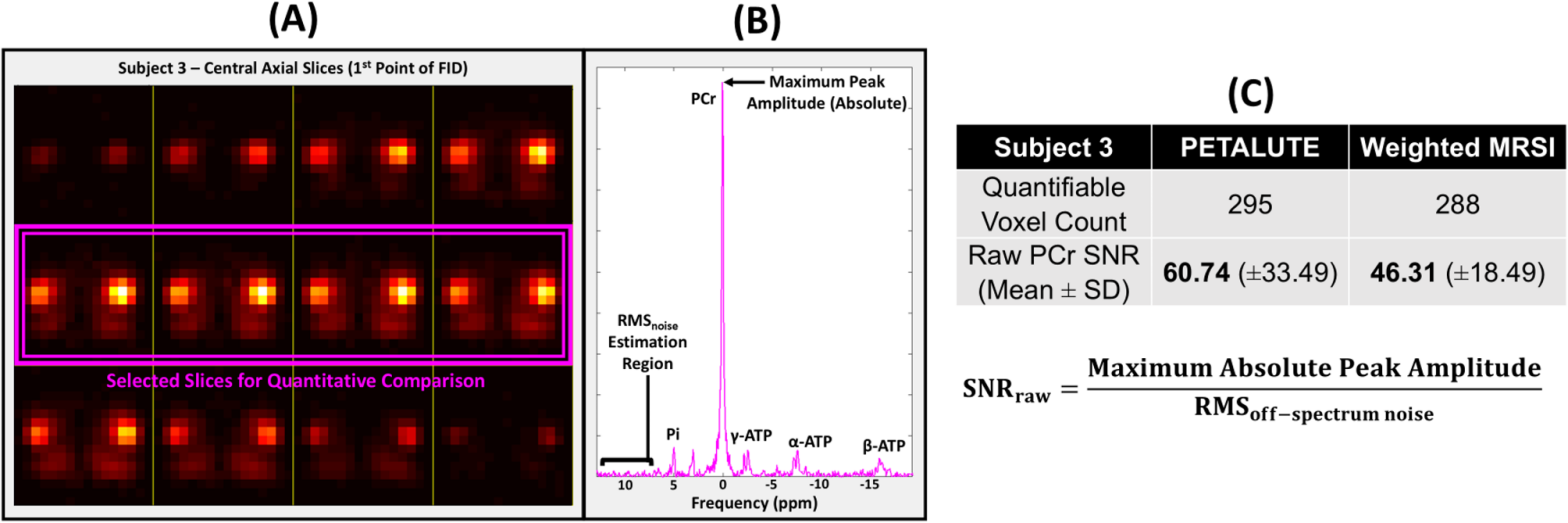
Results from phantom measurements using “raw SNR” (Eq. 4) of absolute inorganic phosphate (Pi) metabolite signal. (**A**) With approximately matched acquisition times, PETALUTE’s mean SNR was 69% higher. (**B**) As both sequences share the same nominal resolution, example axial SNR maps show clear signal intensity across a width of 5 voxels (equivalent to 100 mm). (**C**) A uniform 100-mm diameter Pi bottle phantom was prepared and used for both measurements.

Figure 5 presents results and setup of phantom experiments with dual-tuned flexible surface coil. With approximately matched acquisition times, mean raw SNR (Eq. 4) was 69% higher in PETALUTE than in weighted MRSI. Spectral linewidth was approximately identical between the two acquisitions. Right-tailed t-test confirmed the statistical significance of rosette’s SNR advantage (*p* < 0.001).

### In vivo leg comparison of PETALUTE with weighted MRSI

**Fig. 6:**
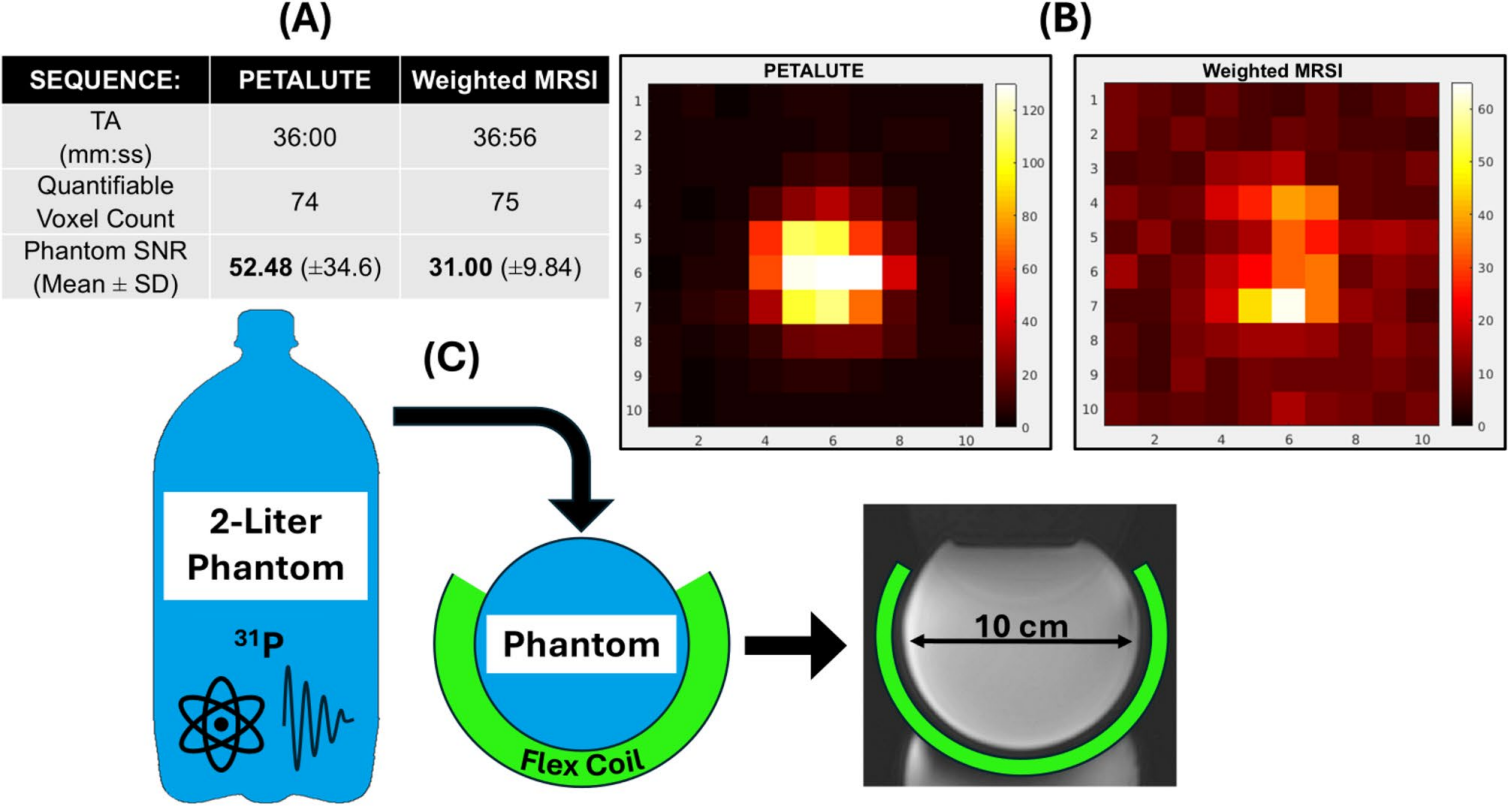
(Left) Example axial slice raw PCr SNR maps from both acquisitions for one quadriceps subject. Both protocols clearly discriminate between high signal muscle tissue and low signal femur region. (Right) Unfiltered magnitude spectra from each method in the highlighted muscle (red) and bone (green) voxels scaled to the maximum PCr peak amplitude. Stated spectral SNR is for PCr peak.

Figure 6 shows representative PETALUTE and weighted MRSI axial PCr maps and spectra in the same volunteer. High-signal muscle regions are clearly distinguishable from low-signal bony femur regions. As expected, PCr predominates the ^31^P muscle spectrum alongside smaller Pi and ATP peaks.

**Fig. 7:**
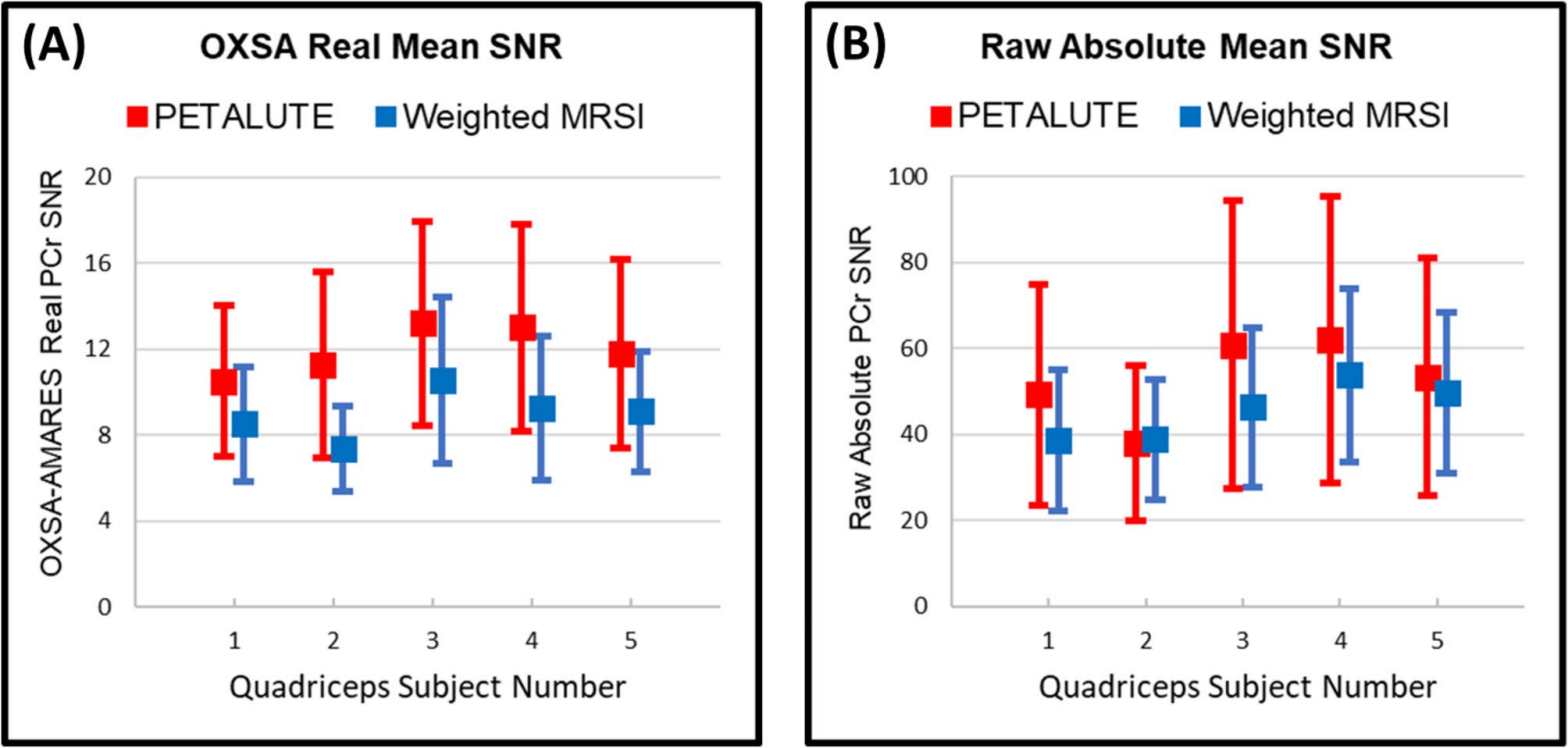
(**A**) Measured PCr SNR (mean ± SD) from OXSA-AMARES quantifiable voxels across all five quadriceps subjects using each acquisition scheme and real data (Eq. 3). By this quantification metric, PETALUTE outperforms weighted MRSI by approximately 34% in vivo. (**B**) Measured raw PCr SNR (mean ± SD) from quantifiable voxels across all five quadriceps subjects and bottle phantom using each acquisition scheme and absolute data (Eq. 4). By this quantification metric, PETALUTE outperformed weighted MRSI by approximately 69% in phantom and 18% in vivo. Detailed results are provided in Tables 2 and 3.

Figure 7 provides the quantitative PCr results for all subjects, illustrating the different SNRs obtained from Eqs. (3) and (4), respectively. Tables 2 and 3, and 4 summarize these distinctions. While PETALUTE consistently outperformed weighted MRSI, the advantage was slightly more prominent in AMARES-fitting of the real data at 34% compared to raw SNR of absolute data at 18%. CRLBs for quantified voxels (see Supplementary Table S1) were well below the 20% limit, with a mean below 3% in all subjects. PCr peak spectral linewidth was nearly matched between the two acquisitions (see Supplementary Table S2) with FWHMs around 4 Hz or below. Right-tailed t-tests confirm the statistical significance of PETALUTE’s higher SNR in both cases (*p* < 0.001).

**Table 2:** Mean in vivo PCr SNR (mean ± SD) from quantifiable voxels in individual subjects using OXSA-AMARES Eq. 3. With matched voxel size and acquisition resolution, PETALUTE outperforms weighted MRSI acquisition in measured SNR by 34% (*p* < 0.001).

	PETALUTE	Weighted MRSI
Subject 1	10.50 (± 3.49)	8.51 (± 2.66)
Subject 2	11.26 (± 4.32)	7.37 (± 1.97)
Subject 3	13.21 (± 4.75)	10.54 (± 3.88)
Subject 4	12.99 (± 4.83)	9.24 (± 3.35)
Subject 5	11.78 (± 4.42)	9.11 (± 2.79)
Total quantified voxels	1070	947
Mean CRLB	2.18%	2.56%
Overall Leg PCr SNR	11.95 (± 4.39)	8.95 (± 3.00)

**Table 3:** Mean in vivo PCr SNR (mean ± SD) from quantifiable voxels in individual subjects using raw Absolute Eq. 4.

	PETALUTE	Weighted MRSI
Subject 1	49.19 (± 25.72)	38.50 (± 16.44)
Subject 2	37.96 (± 17.97)	38.77 (± 14.02)
Subject 3	60.74 (± 33.49)	46.31 (± 18.49)
Subject 4	61.96 (± 33.23)	53.75 (± 20.08)
Subject 5	53.35 (± 27.57)	49.62 (± 18.81)
Total 1uantified voxels	1325	1378
Overall Leg PCr SNR	52.83 (± 28.18)	44.95 (± 17.70)

With matched voxel size and acquisition resolution, PETALUTE outperforms weighted MRSI acquisition in measured SNR by 18% (*p* < 0.001).

**Table 4:** Mean PCr SNR from quantifiable voxels across all five quadriceps subjects and bottle phantom using each method.

	PETALUTE	Weighted MRSI	UTE/MRSI SNR ratio
OXSA-AMARES real in vivo PCr SNR	11.95 (± 4.39)	8.95 (± 3.00)	1.34
Raw absolute in vivo PCr SNR	52.83 (± 28.18)	44.95 (± 17.70)	1.18
Raw absolute Phantom SNR	52.48 (± 34.6)	31.00 (± 9.84)	1.69

With matched voxel size and acquisition resolution, PETALUTE consistently outperforms weighted MRSI acquisition in measured SNR (*p* < 0.001).

## Discussion

### Experimental overview

This study demonstrates the feasibility of using PETALUTE ^31^P-MRSI with a novel rosette k-space trajectory to acquire quality in vivo human subject data. Simulations showed the rosette trajectory produced acceptable image quality and SRF characteristics when compared to a conventional 3D weighted MRSI acquisition. Experimental phantom scans utilized a uniform Pi bottle solution, and the same scanning parameters were later applied to in vivo quadriceps subjects.

### UTE advantages

The novel acquisition’s 70-µs acquisition delay is substantially lower than the 300-500-µs delays in previously published UTE ^31^P-MRSI methods^49,50^, minimizing transverse signal decay and first-order dephasing. Accurate phasing is key to spectral fitting and quantification of real spectral data; when fitting parameters must be tailored to hundreds of voxels across a large volume, such as for high resolution 3D MRSI, the challenge of avoiding phasing errors is most apparent. As expected, all PETALUTE data were intrinsically devoid of noticeable first-order phasing, thereby streamlining the quantification process. The SNR gap between PETALUTE and weighted MRSI acquisitions was narrowed when solely considering absolute data (Eq. 4). This distinction might be partially explained by the absence of phase in these magnitude spectra, whereby the PETALUTE (T_E_ = 70 µs) acquisition loses a portion of its advantage over conventional weighted MRSI (T_E_ = 2.3 ms). Notably, SDs for PETALUTE SNR were significantly higher (50% or more) compared to weighted MRSI. This elevated variation is partially explained by the novel acquisition’s significantly higher SNR; moreover, weighted MRSI’s slightly wider SRF engenders higher inter-voxel crosstalk, diminishing overall variation among quantified voxels.

### Acceleration potential

Although these 36-minute acquisitions are quite lengthy, conventional ungated in vivo 3D ^31^P-MRSI typically requires a minimum of 20 min at 3T. Compared to a weighted Cartesian trajectory, this novel rosette k-space pattern’s relative incoherence makes it a very suitable candidate for CS acceleration via undersampling. Applying undersampling factors of 2 to 4, as demonstrated previously in ultra-short transverse relaxation time (uT_2_) brain imaging^26^ and sodium quantification^30^, could reduce PETALUTE ^31^P-MRSI’s T_A_ to 9–18 min (or less with fewer averages)^57^. Such ongoing investigations^58^ could allow implementation of ^31^P-MRSI within realistic clinical constraints, while also being translatable to UHF research systems and higher resolutions.

As with all ^31^P-MRS, spectral quality can also see potential improvement via proton decoupling and nuclear Overhauser effect (NOE) enhancement, albeit with implications for SAR and measured metabolite ratios^22^. Furthermore, appropriately applied low-rank approximation and principal component analysis denoising have seen use in heightening SNR of MRSI data sets;^59–61^ nevertheless, in the absence of ground truths or precise simulation, care must be taken in estimating metabolite concentration uncertainties after denoising.

### Resolution and SBW

Many non-Cartesian acquisitions face restrictions in spatial resolution, SBW, and SNR due to available gradient hardware^33^. For example, spiral trajectories face reduced SNR while waiting to return to k-space center between spirals; this inefficiency is addressed by closed-loop, out-in trajectories^62^, but these remain impractical outside UHF animal gradient systems. Concentric rings can be similarly adjusted to meet needs with temporal interleaves^43^. Recent work with silent EPSI and gradient insert hardware shows remarkable promise in addressing these constraints^63^.

Clearly, SBW limitations are a significant challenge; although 2.0 kHz might be sufficient for ^31^P-MRS at 3T, such a SBW would only offer a spectral range of around 17 ppm at 7T. While this rosette acquisition sampled 48 points per petal every 480 µs, the sequence remains highly customizable. By leveraging the second half of each petal (Fig. 1), it is possible to partially satisfy Nyquist criterion at even higher bandwidths and enable finer resolution reconstructions than the relatively coarse 8 mL voxels shown here. Additionally, this permits greater SBW acquisitions, opening the door to PETALUTE ^31^P-MRSI at UHF and ^1^H-MRSI at 7T and higher magnetic fields^64^. However, these petal halves are analogous to odd and even echoes of EPSI MRSI; since the timings between individual *N*_*pp*_ are not equidistant, such “full-petal” spectra will suffer from some degree of noise amplification and aliasing artifact.

### Other limitations

Further experimentation is required in exploring the potential and limitations of PETALUTE MRSI. Notably, these scans focused on quadriceps muscle with plentiful PCr signal in a healthy volunteer population. However ^31^, P-MRSI is frequently applied in measuring diverse brain, cardiac, and liver spectra, where nearby tissues may introduce contaminating metabolite signals. Minimal signal contamination was observed in noisy voxels within bony regions. Nonetheless, due to the relative uniformity of skeletal muscle spectra, it would be difficult to discern the rosette acquisition’s relatively incoherent aliasing. As clinical implementation of ^31^P-MRSI necessitates faster acquisition, future work will aim to assess accelerated performance in patient populations.

Notably, these acquisitions did not account for gradient delays in the reconstruction. Since rosette petals gradually evolve from *K*_*z*_ max (a straight line) to *K*_*z*_ = 0 (a round circle), the slew rate and velocity change for petals in different *K*_*z*_ locations. Thus, the gradient delays will have variable impact, introducing an uncertainty in reconstruction which must be better accounted for in rigorous quantitative mapping. Similarly, B_1_-homogeneity can substantially impact measured metabolite signals. While operators performed manual transmit voltage calibration on the vendor adjustment platform before acquisition, variability in B_1_ remains a clear limitation. Future applications should incorporate T_1_/B_1_ mapping to maximize quantitative accuracy across large volumes with multiple metabolites, especially at UHF.

## Conclusions

Using the quadriceps of five healthy volunteers at 3T, we investigated a potential application to ^31^P-MRSI using a novel 3D UTE rosette sequence. In comparison to conventional weighted Cartesian MRSI with matched bandwidth, nominal resolution, and acquisition time, the novel rosette acquisition provided competitive resolution and superior SNR with straightforward quantification. As this proof-of-concept study was limited to five subjects and a relatively homogeneous region of PCr-plentiful muscle, additional testing is required to demonstrate efficacy in differentiating diverse and diseased tissue regions.

## Electronic supplementary material

Below is the link to the electronic supplementary material.

Supplementary Material 1

## Data Availability

Data are available upon reasonable request, by contacting the first author (bbozymsk@purdue.edu).

## References

[1] Ackerman, J. J. H., Grove, T. H., Wong, G. G., Gadian, D. G. & Radda, G. K. Mapping of metabolites in whole animals by 31P NMR using surface coils. *Nature* **283**, 167–170 (1980). 7350541 10.1038/283167a0

[2] Chance, B., Im, J., Nioka, S. & Kushmerick, M. Skeletal muscle energetics with PNMR: Personal views and historic perspectives. *NMR Biomed.* **19**, 904–926 (2006). 17075955 10.1002/nbm.1109

[3] Chance, B., Eleff, S., Leigh, J. S., Sokolow, D. & Sapega, A. Mitochondrial regulation of phosphocreatine/inorganic phosphate ratios in exercising human muscle: A gated 31P NMR study. *Proc. Natl. Acad. Sci. U S A*. **78**, 6714 (1981). 6947247 10.1073/pnas.78.11.6714 PMC349120

[4] Podo, F. Tumour phospholipid metabolism. 10.1002/(SICI)1099-1492(199911)12:7 (1999). 10.1002/(sici)1099-1492(199911)12:7<413::aid-nbm587>3.0.co;2-u 10654290

[5] Cox, I. J. et al. In vivo and in vitro 31P magnetic resonance spectroscopy of focal hepatic malignancies. *NMR Biomed.* **5**, 114–120 (1992). 1322688 10.1002/nbm.1940050303

[6] Bell, J. D. & Bhakoo, K. K. metabolic changes underlying 31 P MR spectral alterations in human hepatic tumours. 10.1002/(SICI)1099-1492(1998110)11:7 (1998). 10.1002/(sici)1099-1492(1998110)11:7<354::aid-nbm515>3.0.co;2-n 9859941

[7] Dagnelie, P. C. et al. Abnormal liver metabolism in cancer patients detected by 31 P MR spectroscopy. 10.1002/(SICI)1099-1492(199912)12:8 (1999). 10.1002/(sici)1099-1492(199912)12:8<535::aid-nbm601>3.0.co;2-1 10668046

[8] Dagnelie, P. C. & Leij-Halfwerk, S. Magnetic resonance spectroscopy to study hepatic metabolism in diffuse liver diseases, diabetes and cancer. *World J. Gastroenterol.: WJG* **16**, 1577 (2010). 20355236 10.3748/wjg.v16.i13.1577 PMC2848366

[9] Glazer, G. M. et al. Image localized 31P magnetic resonance spectroscopy of the human liver. *NMR Biomed.* **1**, 184–189 (1989). 2561877 10.1002/nbm.1940010406

[10] Seelen, L. W. F. et al. Prospective of 31 P MR Spectroscopy in Hepatopancreatobiliary Cancer: a systematic review of the literature. *J. Magn. Reson. Imaging* **57**, 1144–1155 (2023). 35916278 10.1002/jmri.28372

[11] Scheuermann-Freestone, M. et al. Abnormal cardiac and skeletal muscle energy metabolism in patients with type 2 diabetes. *Circulation* **107**, 3040–3046 (2003). 12810608 10.1161/01.CIR.0000072789.89096.10

[12] Jett, S. et al. Systematic review of 31P-magnetic resonance spectroscopy studies of brain high energy phosphates and membrane phospholipids in aging and Alzheimer’s disease. *Front. Aging Neurosci.* **15**, 1183228 (2023). 10.3389/fnagi.2023.1183228 PMC10232902 37273652

[13] Tsampasian, V., Cameron, D., Sobhan, R., Bazoukis, G. & Vassiliou, V. S. Phosphorus magnetic resonance spectroscopy (31P MRS) and cardiovascular disease: The importance of energy. *Med. (Kaunas)* **59**(1), 174 (2023). 10.3390/medicina59010174 PMC9866867 36676798

[14] Rider, O. J. et al. Effects of catecholamine stress on diastolic function and myocardial energetics in obesity. *Circulation* **125**, 1511–1519 (2012). 22368152 10.1161/CIRCULATIONAHA.111.069518

[15] Levelt, E. et al. Cardiac energetics, oxygenation, and perfusion during increased workload in patients with type 2 diabetes mellitus. *Eur. Heart J.* **37**, 3461–3469 (2016). 26392437 10.1093/eurheartj/ehv442 PMC5201143

[16] Maris, J. M. et al. 31P nuclear magnetic resonance spectroscopic investigation of human neuroblastoma in situ. *N Engl. J. Med.* **312**, 1500–1505 (1985). 3990750 10.1056/NEJM198506063122307

[17] Pinggera, D. et al. Repeated 31P-Magnetic resonance spectroscopy in severe traumatic brain Injury: insights into cerebral energy status and altered metabolism. *J. Neurotrauma* **38**, 2822–2830 (2021). 34235953 10.1089/neu.2021.0143

[18] Gadian, D. G., Dawson, M. J. & Wilkie, D. R. Contraction and recovery of living muscles studies by 31P nuclear magnetic resonance. *J. Physiol.* **267**, 703–735 (1977). 17739 10.1113/jphysiol.1977.sp011835 PMC1283637

[19] Ross, B. D. et al. Examination of a case of suspected McArdle’s syndrome by 31P nuclear magnetic resonance. *N Engl. J. Med.* **304**, 1338–1342 (1981). 6938778 10.1056/NEJM198105283042206

[20] Valkovič, L., Chmelík, M. & Krššák, M. In-vivo31P-MRS of skeletal muscle and liver: A way for non-invasive assessment of their metabolism. *Anal. Biochem.* **529**, 193–215 (2017). 28119063 10.1016/j.ab.2017.01.018 PMC5478074

[21] Meyerspeer, M., Krššák, M. & Moser, E. Relaxation times of 31P-metabolites in human calf muscle at 3 T. *Magn. Reson. Med.* **49**, 620–625 (2003). 12652531 10.1002/mrm.10426

[22] Meyerspeer, M. et al. 31 P magnetic resonance spectroscopy in skeletal muscle: Experts’ consensus recommendations. *NMR Biomed.* **34**(5), e4246 (2020). 10.1002/nbm.4246 PMC8243949 32037688

[23] Jonuscheit, M. et al. Reproducibility of absolute quantification of adenosine triphosphate and inorganic phosphate in the liver with localized 31P-magnetic resonance spectroscopy at 3-T using different coils. *NMR Biomed.* **37** (8), e5120 (2024). 38404058 10.1002/nbm.5120

[24] Rodgers, C. T. et al. Human cardiac 31P magnetic resonance spectroscopy at 7 tesla. *Magn. Reson. Med.* **72**, 304 (2014). 24006267 10.1002/mrm.24922 PMC4106879

[25] Noll, D. C. Multishot rosette trajectories for spectrally selective mr imaging. *IEEE Trans. Med. Imaging* **16**, 372–377 (1997). 9262995 10.1109/42.611345

[26] Shen, X. et al. Ultra-short T2 components imaging of the whole brain using 3D dual-echo UTE MRI with rosette k-space pattern. *Magn. Reson. Med.* **89**, 508–521 (2023). 36161728 10.1002/mrm.29451 PMC9712161

[27] Shen, X. et al. 3D balanced SSFP UTE MRI for multiple contrasts whole brain imaging. *Magn. Reson. Med.* **92**, 702–714 (2024). 38525680 10.1002/mrm.30093

[28] Shen, X. et al. High-resolution 3D ultra-short echo time MRI with Rosette k-space pattern for brain iron content mapping. *J. Trace Elem. Med. Biol.* **77**, 127146 (2023). 10.1016/j.jtemb.2023.127146 PMC10107748 36871432

[29] Monsivais, H. et al. Ultrashort-echo time magnetization transfer (UTE-MT) for brain iron imaging. in *Presented During ISMRM Annual Meeting & Exhibition; June 3–8, 2023; Toronto, CA*.

[30] Villarreal, C. X. et al. An Accelerated PETALUTE MRI sequence for in vivo quantification of sodium content in human articular cartilage at 3T. *Skeletal Radiol* (2024). 10.1007/s00256-024-04774-5 PMC11769871 39153083

[31] Bozymski, B. et al. Comparison of compressed sensing accelerated rosette UTE and conventional 31P 3D MRSI at 3T in leg muscle. in *Presented During ISMRM Annual Meeting & Exhibition; June 3–8, 2023; Toronto, CA*.

[32] Li, Y. et al. Analysis of generalized rosette trajectory for compressed sensing MRI. *Med. Phys.* **42**, 5530–5544 (2015). 26329000 10.1118/1.4928152

[33] Bogner, W., Otazo, R. & Henning, A. Accelerated MR spectroscopic imaging: A review of current and emerging techniques. *NMR Biomed.* **34**, e4314 (2021). 32399974 10.1002/nbm.4314 PMC8244067

[34] Bakermans, A. J. et al. In vivo mouse myocardial (31)P MRS using three-dimensional image-selected in vivo spectroscopy (3D ISIS): Technical considerations and biochemical validations. *NMR Biomed.* **28**, 1218–1227 (2015). 26269430 10.1002/nbm.3371 PMC4573916

[35] de Wit-Verheggen, V. H. W. et al. PCr/ATP ratios and mitochondrial function in the heart: A comparative study in humans. *Sci. Rep.* **13**, 8346 (2023). 37221197 10.1038/s41598-023-35041-7 PMC10205750

[36] Korzowski, A. & Bachert, P. High-resolution 31 P echo-planar spectroscopic imaging in vivo at 7T. *Magn. Reson. Med.* **79**, 1251–1259 (2018). 28639310 10.1002/mrm.26785

[37] Cunningham, C. H. et al. Design of flyback echo-planar readout gradients for magnetic resonance spectroscopic imaging. *Magn. Reson. Med.* **54**, 1286–1289 (2005). 16187273 10.1002/mrm.20663

[38] Santos-Díaz, A., Obruchkov, S. I., Schulte, R. F. & Noseworthy, M. D. Phosphorus magnetic resonance spectroscopic imaging using flyback echo planar readout trajectories. *MAGMA* **31**, 553–564 (2018). 29383517 10.1007/s10334-018-0675-y

[39] Santos-Díaz, A., Harasym, D. & Noseworthy, M. D. Dynamic 31 P spectroscopic imaging of skeletal muscles combining flyback echo-planar spectroscopic imaging and compressed sensing. *Magn. Reson. Med.* **81**, 3453–3461 (2019). 30737840 10.1002/mrm.27682

[40] Posse, S., Tedeschi, G., Risinger, R., Ogg, R. & Bihan, D. Le. High speed 1H spectroscopic imaging in human brain by echo planar spatial-spectral encoding. *Magn. Reson. Med.* **33**, 34–40 (1995). 7891533 10.1002/mrm.1910330106

[41] Cunningham, C. H. et al. Pulse sequence for dynamic volumetric imaging of hyperpolarized metabolic products. *J. Magn. Reson.* **193**, 139–146 (2008). 18424203 10.1016/j.jmr.2008.03.012 PMC3051833

[42] Nam, K. M. et al. Deuterium echo-planar spectroscopic imaging (EPSI) in the human liver in vivo at 7 T. *Magn. Reson. Med.* **90**, 863–874 (2023). 37154391 10.1002/mrm.29696

[43] Clarke, W. T. et al. Three-dimensional, 2.5-minute, 7T phosphorus magnetic resonance spectroscopic imaging of the human heart using concentric rings. *NMR Biomed.* **36**(1), e4813 (2023). 10.1002/nbm.4813 PMC7613900 35995750

[44] Furuyama, J. K., Wilson, N. E. & Thomas, M. A. Spectroscopic imaging using concentrically circular echo-planar trajectories in vivo. *Magn. Reson. Med.* **67**, 1515–1522 (2012). 22006586 10.1002/mrm.23184

[45] Adalsteinsson, E. et al. Volumetric spectroscopic imaging with spiral-based k-space trajectories. *Magn. Reson. Med.* **39**, 889–898 (1998). 9621912 10.1002/mrm.1910390606

[46] Valkovič, L. et al. Dynamic 31P–MRSI using spiral spectroscopic imaging can map mitochondrial capacity in muscles of the human calf during plantar flexion exercise at 7 T. *NMR Biomed.* **29**, 1825 (2016). 27862510 10.1002/nbm.3662 PMC5132121

[47] Ludwig, D., Korzowski, A., Ruhm, L., Ladd, M. E. & Bachert, P. Three-dimensional 31P radial echo-planar spectroscopic imaging in vivo at 7T. in *Presented during ISMRM 25th Annual Meeting and Exhibition; April 22–27, 2017; Honolulu, HI*.

[48] Robson, M. D., Tyler, D. J. & Neubauer, S. Ultrashort TE Chemical Shift Imaging (UTE-CSI). *Magn. Reson. Med.* **53**, 267–274 (2005). 15678544 10.1002/mrm.20344

[49] Tyler, D. J. et al. Reproducibility of 31P cardiac magnetic resonance spectroscopy at 3 T. *NMR Biomed.* **22**, 405–413 (2009). 19023865 10.1002/nbm.1350

[50] Ellis, J., Valkovič, L., Purvis, L. A. B., Clarke, W. T. & Rodgers, C. T. Reproducibility of human cardiac phosphorus MRS (31P-MRS) at 7 T. *NMR Biomed.* **32**(6), e4095 (2019). 10.1002/nbm.4095 PMC6546607 30924566

[51] Fessler, J. & Sutton, B. Nonuniform fast Fourier transforms using min-max interpolation. *IEEE Trans. Signal Process.* **51**, 560–574 (2003).

[52] Panda, A. et al. Phosphorus liver MRSI at 3 T using a novel dual-tuned eight-channel ^31^P/^1^H H coil. *Magn. Reson. Med.* **68**, 1346–1356 (2012). 22287206 10.1002/mrm.24164

[53] Fessler, J. A. On NUFFT-based gridding for non-cartesian MRI. *J. Magn. Reson.* **188**, 191–195 (2007). 17689121 10.1016/j.jmr.2007.06.012 PMC2121589

[54] Pipe, J. G. & Menon, P. Sampling density compensation in MRI: Rationale and an iterative numerical solution. *Magn. Reson. Med.* **41**, 179–186 (1999). 10025627 10.1002/(sici)1522-2594(199901)41:1<179::aid-mrm25>3.0.co;2-v

[55] Rodgers, C. T. & Robson, M. D. Receive array magnetic resonance spectroscopy: Whitened singular value decomposition (WSVD) gives optimal bayesian solution. *Magn. Reson. Med.* **63**, 881–891 (2010). 20373389 10.1002/mrm.22230

[56] Purvis, L. A. B. et al. An open-source magnetic resonance spectroscopy analysis toolbox in MATLAB. *PLoS One* **12**, e0185356 (2017). 28938003 10.1371/journal.pone.0185356 PMC5609763

[57] Farley, N., Bozymski, B., Dydak, U. & Emir, U. Fast 3D-P31-MRSI using custom rosette petal trajectory at 3T with 4x accelerated compressed sensing. in *Presented during ISMRM Annual Meeting & Exhibition; June 3–8; Toronto, CA* (2023).

[58] Alcicek, S. et al. Multi-site ultrashort echo time 3D Phosphorous MRSI repeatability using novel rosette trajectory (PETALUTE). bioRxiv 10.1101/2024.02.07.579294 (2024).

[59] Nguyen, H. M., Peng, X., Do, M. N. & Liang, Z. P. Denoising MR spectroscopic imaging data with low-rank approximations. *IEEE Trans. Biomed. Eng.* **60**, 78–89 (2013). 23070291 10.1109/TBME.2012.2223466 PMC3800688

[60] Clarke, W. T. & Chiew, M. Uncertainty in denoising of MRSI using low-rank methods. *Magn. Reson. Med.* **87**, 574–588 (2022). 34545962 10.1002/mrm.29018 PMC7612041

[61] Froeling, M., Prompers, J. J., Klomp, D. W. J. & van der Velden, T. A. PCA denoising and Wiener deconvolution of 31P 3D CSI data to enhance effective SNR and improve point spread function. *Magn. Reson. Med.* **85**, 2992–3009 (2021). 33522635 10.1002/mrm.28654 PMC7986807

[62] Esmaeili, M. et al. Whole-slab 3D MR spectroscopic Imaging of the human brain with spiral-out-In sampling at 7T. *J. Magn. Reson. Imaging* **53**, 1237–1250 (2021). 33179836 10.1002/jmri.27437 PMC8717862

[63] Versteeg, E. et al. A silent echo-planar spectroscopic imaging readout with high spectral bandwidth MRSI using an ultrasonic gradient axis. *Magn. Reson. Med.* **91**, 2247–2256 (2024). 38205917 10.1002/mrm.30008

[64] Emir, U. E., Sawiak, S. Bridging the gap between Preclinical, Clinical, Metabolomics, MRI, MRS(I) methods via PETALUTE MRI. Presented at ISMRM Workshop on MR Spectroscopy: Frontiers in Molecular & Metabolic Imaging, Boston (2024).

