# Supplementary material for: Feasibility and comparison of 3D modified rosette ultra-short echo time (PETALUTE) with conventional weighted acquisition in 31P-MRSI
Source: Sci Rep. 2025 Feb 22;15:6465. doi: 10.1038/s41598-025-90630-y (PMC11846993; doi:10.1038/s41598-025-90630-y)
Supplement: Supplementary file 1 — Supplementary Material 1 [file 41598_2025_90630_MOESM1_ESM.docx]

**Supplementary Materials**

|  | **PETALUTE Voxels** | **PETALUTE CRLB (%)** | **Weighted MRSI Voxels** | **Weighted MRSI CRLB (%)** |
| --- | --- | --- | --- | --- |
| Subject 1 | 229 | 2.37 (±0.51) | 230 | 2.81 (±0.90) |
| Subject 2 | 205 | 2.17 (±0.55) | 168 | 2.98 (±0.88) |
| Subject 3 | 227 | 2.17 (±1.04) | 194 | 2.27 (±0.80) |
| Subject 4 | 219 | 1.98 (±0.55) | 185 | 2.38 (±0.95) |
| Subject 5 | 190 | 2.17 (±0.59) | 170 | 2.34 (±0.87) |
| **Overall** | **1070** | **2.18 (±0.68)** | **947** | **2.56 (±0.88)** |

**Supplementary Table S1** Overview of quantified voxels and associated CRLBs (mean ± SD) for PCr SNR analysis in individual subjects using OXSA-AMARES Equation (3). CRLBs for all voxels were well below the 20% limit, with a mean below 3% in all subjects.

|  | **PETALUTE Linewidth (Hz)** | **Weighted MRSI Linewidth (Hz)** |
| --- | --- | --- |
| Subject 1 | 3.75 (±0.12) | 3.62 (±0.12) |
| Subject 2 | 3.85 (±0.17) | 3.56 (±0.08) |
| Subject 3 | 3.92 (±0.24) | 3.67 (±0.18) |
| Subject 4 | 3.90 (±0.20) | 3.59 (±0.12) |
| Subject 5 | 3.85 (±0.21) | 3.61 (±0.12) |
| **Overall** | **3.86 (±0.19)** | **3.61 (±0.13)** |

**Supplementary Table S2** Comparison of PCr spectral linewidth (mean ± SD) for quantified voxels in individual subjects. Reported value is the full width at half maximum (FWHM) of the peak, obtained from the best fitting Lorentzian function. Linewidths are nearly matched, with PETALUTE mean FWHM exceeding that of weighted MRSI by 6.9%.
